# Is Doppler ultrasound useful for evaluating gestational trophoblastic disease?

**DOI:** 10.6061/clinics/2015(12)08

**Published:** 2015-12

**Authors:** Lawrence H Lin, Lisandra S Bernardes, Eliane A Hase, Koji Fushida, Rossana P V Francisco

**Affiliations:** Faculdade de Medicina da Universidade de São Paulo, Departamento de Obstetrícia e Ginecologia, Centro de Doenças Trofoblásticas, São Paulo/SP, Brazil.

**Keywords:** Gestational Trophoblastic Disease, Trophoblastic Neoplasms, Hydatidiform Mole, Ultrasonography, Doppler, Neovascularization, Pathologic

## Abstract

Doppler ultrasound is a non-invasive method for evaluating vascularization and is widely used in clinical practice. Gestational trophoblastic neoplasia includes a group of highly vascularized malignancies derived from placental cells. This review summarizes data found in the literature regarding the applications of Doppler ultrasound in managing patients with gestational trophoblastic neoplasia.

The PubMed/Medline, Web of Science, Cochrane and LILACS databases were searched for articles published in English until 2014 using the following keywords: “Gestational trophoblastic disease AND Ultrasonography, Doppler.”

Twenty-eight articles met the inclusion criteria and were separated into the 4 following groups according to the aim of the study. 1 Doppler ultrasound does not seem to be capable of differentiating partial from complete moles, but it might be useful when evaluating pregnancies in which a complete mole coexists with a normal fetus. 2 There is controversy in the role of uterine artery Doppler velocimetry in the prediction of development of gestational trophoblastic neoplasia. 3 Doppler ultrasound is a useful tool in the diagnosis of gestational trophoblastic neoplasia because abnormal myometrial vascularization and lower uterine artery Doppler indices seem to be correlated with invasive disease. 4 Lower uterine artery Doppler indices in the diagnosis of gestational trophoblastic neoplasia are associated with methotrexate resistance and might play a role in prognosis.

**CONCLUSION:**

Several studies support the importance of Doppler ultrasound in the management of patients with gestational trophoblastic neoplasia, particularly the role of Doppler velocimetry in the prediction of trophoblastic neoplasia and the chemoresistance of trophoblastic tumors. Doppler findings should be used as ancillary tools, along with human chorionic gonadotropin assessment, in the diagnosis of gestational trophoblastic neoplasia.

## INTRODUCTION

Gestational trophoblastic disease includes a spectrum of conditions characterized by abnormal trophoblast proliferation and invasion. It primarily comprises five diseases: hydatidiform mole, invasive mole, choriocarcinoma, placental site trophoblastic tumor and epithelioid trophoblastic tumor. Hydatidiform mole is the most common form, with an estimated incidence of 1 case in 1,000 pregnancies. Although it is generally a benign condition, it can evolve into an invasive form in some patients. The remaining diseases are all malignant forms termed gestational trophoblastic neoplasia (GTN) or gestational trophoblastic tumors. These tumors are highly vascularized and, in the past, were often evaluated by arteriography 1.

Doppler ultrasonography is a noninvasive method currently used to reliably evaluate and measure blood flow. Doppler ultrasound includes color, power and spectral modalities. Color flow imaging evaluates the direction of blood flow and is based on a frequency change. In contrast, power Doppler is based on amplitude changes and is thus more sensitive to slow velocity flows but does not provide direction information. Spectral Doppler provides a functional assessment of the circulation by calculating Doppler velocimetry indices based on the waveforms of blood flow. The most commonly used Doppler indices are the ratio of peak systolic to end diastolic velocity (S/D), resistance index (RI; calculated as [peak systolic velocity - end diastolic velocity / peak systolic velocity]) and pulsatility index (PI, calculated as [peak systolic velocity - end diastolic velocity / mean velocity]) 2.

It has been shown that Doppler ultrasonography presents almost the same findings (tumor size and localization) as a pelvic arteriogram in women with GTN and this approach is currently widely used and substituted arteriography in the evaluation of such patients 1,3. Below, we summarize the main purposes of Doppler sonography in the management of patients with gestational trophoblastic disease.

## METHODS

The PubMed/Medline, Web of Science, Cochrane and LILACS databases were searched for articles published in English until 2014 using the following Medical Subject Heading (MeSH) keywords: “Gestational trophoblastic disease AND Ultrasonography, Doppler.” Studies evaluating the role of Doppler ultrasound in the diagnosis, prognosis and follow-up of patients with gestational trophoblastic disease were included. Case reports, reviews, letters to the editor and articles not in English were excluded. All abstracts were analyzed and included according to the current study criteria. The selected articles’ bibliographies were also evaluated and the relevant references were included.

## RESULTS AND DISCUSSION

**Figure 1:**
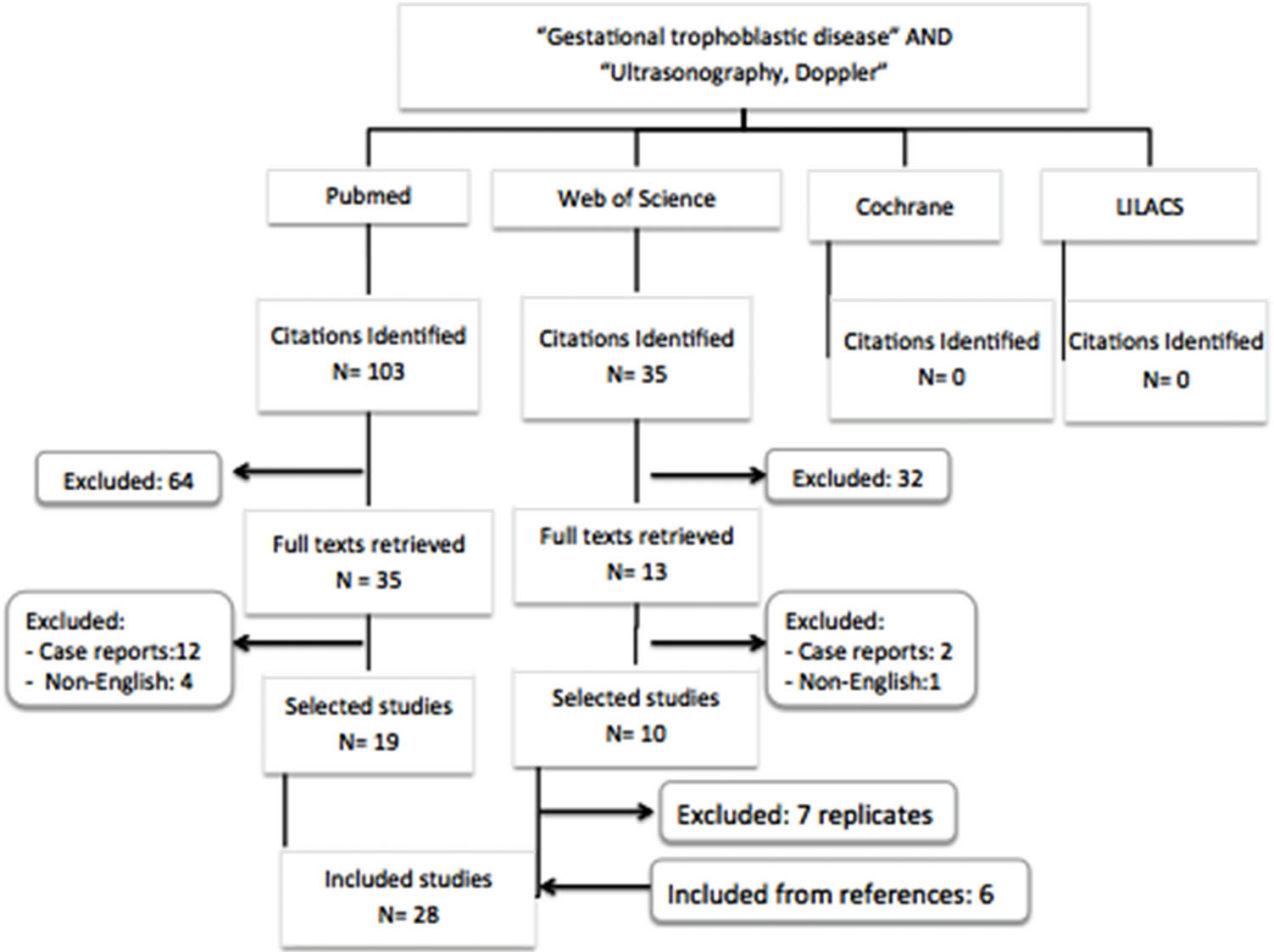
Flow chart describing the mechanism of search.

The initial search identified 103 articles in PubMed, 35 in Web of Science, and no articles in the Cochrane and LILACS databases. Twenty-eight articles were selected for this review according to the inclusion and exclusion criteria (Figure 1). The articles were divided into 4 groups according to the aim of the study.

### Doppler evaluation for the differentiation of partial and complete moles

Differentiating between partial and complete moles carries prognostic significance because of the higher rate of post-molar disease in complete moles (15-20%) compared with partial moles (less than 5%). In most cases, ultrasonography can identify the type of mole. However, in certain situations (such as a complete mole with a coexisting normal fetus), ancillary methods such as Doppler ultrasound are useful.

In a retrospective study, a higher RI of the uterine arteries (UA) was reported in normal pregnancies (n=13; mean RI=0.66±0.05) compared with molar pregnancies, but there was no significant difference between the UA RI of partial moles (n=33; mean RI=0.56±0.04) and complete moles (n=106; mean RI=0.55±0.06) 4. Jauniaux et al. (1996) described a series of 70 cases of triploidy after the first trimester and demonstrated that at least half of the cases with a UA evaluation (n=41) presented with an abnormally high RI 5. In a series of cases presented by Jauniaux & Nicolaides, the UA PI after 16 weeks was below the fifth percentile in all three complete moles that coexisted with a normal fetus, in contrast with a uterine artery PI within normal ranges in six cases of partial moles 6.

Trophoblastic cells at the implantation site of each type of pregnancy seem to differ according to the level of invasiveness observed in the first trimester of molar pregnancies, particularly in complete moles 7, which might explain the difference in Doppler velocimetry of the UA. In clinical practice, the values of the UA Doppler indices do not seem to help differentiate between partial and complete moles, except when complete moles coexist with a normal fetus.

### Doppler ultrasound in the prediction of GTN

There are few predictors of the clinical course of hydatidiform moles. Advanced maternal age, higher human chorionic gonadotropin (hCG) levels, larger uterine size, complete moles, large theca lutein cysts and clinical complications have been associated with malignant post-molar disease. Few studies have evaluated the role of Doppler ultrasound in the prediction of GTN development.

Lower UA Doppler indices before molar evacuation were associated with the development of GTN in 5 patients (mean S/D=5.10; mean RI=0.80; mean PI=1.82) compared with 16 patients who exhibited spontaneous remission (mean S/D=2.27; mean RI=0.55; mean PI=0.86) 8. Similarly, Gungor et al. (1998) reported a lower UA RI before molar evacuation in 12 patients who developed persistent malignant disease (mean RI=0.29) compared to 20 patients with spontaneous remission (mean RI=0.46) 9. In contrast, the study by Chan et al. (1996) showed no difference in the UA RI in 11 patients with spontaneous remission compared with 21 patients with post-molar disease 10. A preliminary study that included 8 patients reported that a lower UA PI (<1.5) after 2 weeks of evacuation was only observed in the 2 patients with GTN prior to the hCG increase 11.

Doppler ultrasound seems to play a role in predicting GTN following uterine evacuation. In a retrospective cohort, the presence of Doppler ultrasound abnormalities, such as nodules or hypervascularization in the myometrium or endometrium, after 3 weeks of molar evacuation was highly correlated with the development of GTN in 14 patients compared to 175 patients who exhibited spontaneous remission 12. Zanetta et al. 13 evaluated the efficiency of uterine morphology and Doppler ultrasound in the diagnosis of post-molar disease. The Doppler results were considered abnormal when at least three vessels in the myometrium or residual intrauterine tissue had a PI below 1.0. In 16 patients with spontaneous regression of hCG, the PI values showed progressive increases, and the vascularization was reduced during follow-up. Normal Doppler findings had a negative predictive value of 100% for persistent disease, and normalization of the findings occurred up to 8 weeks before hCG became undetectable. In contrast, an abnormal Doppler at the 6^th^ week post-evacuation showed a positive predictive value of 67% for local persistence. In a follow-up of 9 patients with GTN, only one patient presented with normal ultrasound findings and elevated hCG, which was related to extra-uterine metastasis 13.

Although most studies have noted that a lower resistance in the UA is associated with the development of trophoblastic tumors (Table 1), data in the literature remain controversial. New studies with larger samples are thus needed to better elucidate the role of Doppler ultrasound in predicting post-molar disease.

### Post-molar follow-up and diagnosis of trophoblastic neoplasia

Following uterine molar evacuation, patients are followed closely with weekly clinical evaluations and hCG monitoring. An ultrasound is only generally needed when there is an increase or plateau in hCG titers when evaluating a primary GTN site. Using Doppler ultrasonography to detect post-molar disease was found to be more efficient, primarily in microscopic disease compared with standard ultrasound scans, in a series of patients with persistent trophoblastic disease who underwent dilation and curettage 14.

Emoto & Sadamori 15 described the presence of intratumoral blood flow by color Doppler ultrasonography in all forms of trophoblastic tumors (5 invasive moles, 1 choriocarcinoma and 1 placental site trophoblastic tumor), whereas this observation was not made in 15 hydatidiform moles. Moreover, after contrast enhancement using a Levovist microbubble contrast agent, there was a significant increase in flow in the small invasive moles (<2 cm) 15.

A large retrospective study of 355 patients with trophoblastic disease reported a higher UA RI in normal pregnancies (n=13; mean RI=0.66), followed by partial (n=33; RI=0.56) and complete hydatidiform moles (n=106; mean RI=0.55), compared to GTN pregnancies (mean RI=0.28 and RI=0.25 for 184 presumed invasive moles and 32 choriocarcinomas, respectively) 4. In other studies, it was shown that patients with trophoblastic tumors had a lower resistance on UA Doppler than did those with a hydatidiform mole or women who were post-abortion, non-pregnant or in the first trimester of pregnancy (Taylor et al. 16; Long et al. 1990; Tepper et al. 18; Chan et al. 10; Hsieh et al. 1994) (Table 2).

There is also evidence of a strong correlation between UA Doppler indices and hCG regression patterns in hydatidiform moles and post-molar tumors, with a concurrent increase in the Doppler indices and a decrease in hCG after uterine evacuation 8,10,13,18,20,21.

The blood flow area ratio (BFAR) is a way to quantify blood flow in an area of interest using appropriate software based on 2D power Doppler sonography. The BFAR in 19 patients with invasive trophoblastic disease was significantly higher than in 25 women with a normal pregnancy or 25 non-pregnant individuals. It was also demonstrated that the BFAR gradually decreased during and after chemotherapy, but it took 3 to 6 months after chemotherapy to normalize in value 22.

Hsieh et al. 23 described three different vascular patterns for Doppler ultrasound in 28 trophoblastic tumors: diffuse, lacunar and compact. These patterns showed different UA RI (more compact and fewer lacunar type), hCG levels (more compact and fewer diffuse type) and the need for more than 5 chemotherapy cycles (more compact and fewer diffuse type). Histopathological analysis was available in 11 cases; of these, 7 invasive moles were the lacunar type, and 4 choriocarcinomas were the compact type, which indicated that on some level, Doppler ultrasound is capable of differentiating trophoblastic tumors 23.

Doppler ultrasound seems to be a useful adjuvant tool in post-molar follow-up and GTN diagnosis because it has been well-demonstrated in the literature that abnormal Doppler findings evaluated using different methods (such as abnormal myometrial vascularization and lower UA Doppler indices) are correlated with invasive disease. Increased vascularization of the myometrium, higher UA peak systolic velocity, and lower UA RI and PI were all associated with GTN, although the Doppler indices varied between studies (Table 2). Additional useful information for follow-up includes an inverse correlation between UA Doppler indices and hCG regression. However, some caution should be taken when analyzing abnormal Doppler images alone after uterine evacuation or chemotherapy because of the development and persistence of arteriovenous communications in the myometrium; in these cases, the hCG level is normal 13.

### Prediction of resistance to chemotherapy

Several studies have demonstrated a rough correlation between uterine Doppler vascularization and the response to chemotherapy 3,24. A pilot study assessed ultrasound and Doppler signs of response (decreases in tumor size, echogenicity and abnormal myometrial Doppler signal) in seven patients with chemoresistance to methotrexate suspected by an hCG plateau. Three of these patients had ultrasound findings that indicated a response and achieved remission after methotrexate. This study suggests that an ultrasound can differentiate patients with a delayed response to methotrexate from those who require second-line treatment 25.

An increase in the UA Doppler indices of patients responding to chemotherapy was observed in parallel with a decrease in hCG levels 18. Although the initial UA S/D ratio was not significantly different between the 16 patients with chemoresistant (2.69±1.8) and chemosensitive disease (2.72±1.31), after the completion of chemotherapy, the UA S/D ratio increased only in patients with remitted disease (6.23±2.38). However, this was not observed in patients with non-remitted disease (3.08±1.54) 26.

Hsieh et al. 19 described a higher mean UA RI in 10 patients requiring fewer than 5 cycles of chemotherapy (mean RI=0.71±0.09) compared with 13 patients who required longer chemotherapy (mean RI=0.47±0.14). In addition, there was an evident decrease in the peak systolic velocity after the first three chemotherapy cycles in the group that required fewer courses of treatment (54.2 to 23.6) compared to the other group (60.1 to 60) 23. These findings indicate a greater change in uterine hemodynamics in patients with a better response to chemotherapy, which could help in the decision to change chemotherapy.

Deeper myometrial invasion and lower RI from myometrial vascularization were correlated with methotrexate resistance in 37 patients with GTN. Thirty patients showed remission with a single chemotherapy agent, whereas 7 patients required an actinomycin D combination chemotherapy or hysterectomy. A cuff-off value of 4 cm or higher for myometrial invasion (sensibility 100% and specificity 75%) and a myometrial vascularization RI≤0.28 (sensibility 87.5 and specificity 83.3) were proposed to predict single-agent chemotherapy resistance 27.

Several studies have evaluated the prediction of methotrexate resistance with UA PI prior to chemotherapy. Long et al. 17 prospectively evaluated the UA PI of 40 patients with GTN and proposed a PI cut-off value of ≤1.1 for inclusion in the prognostic scoring system of trophoblastic tumors due to the elevated likelihood of these patients developing chemoresistance 28. In a retrospective study of 164 patients, the UA PI was an independent factor for methotrexate resistance. A PI≤1.0 detected resistance with a sensitivity of 68% and specificity of 62%, with an adjusted odds ratio of 2.27 for methotrexate resistance. Furthermore, 72.2% of the patients with a Charing Cross Hospital prognostic score between 6 and 8 and a PI≤1 developed resistance. In contrast, only 20% of the patients with a PI>1 developed resistance 29. Similarly, the UA PI was identified as an important factor associated with methotrexate resistance in 73 patients with a GTN FIGO score of 5-6. A chemoresistance risk of 67% was calculated for patients with UA PI≤1, compared to 42% when the UA PI was >1 (*p*=0.036) 30. Another study that assessed the UA PI and methotrexate resistance in 239 patients with a FIGO score of 0-6 showed a lower UA PI in patients with chemoresistance and found that a UA PI≤1 served as a risk factor, independent of the FIGO scoring system for methotrexate resistance. Patients with a FIGO score of 6 and UA PI≤1 showed 100% resistance to methotrexate compared with 20% of the patients who had a UA PI>1. The authors suggest that these patients might benefit from upstaging and combination therapy 31.

Numerous studies support the utilization of Doppler ultrasound as a tool to predict chemotherapy resistance (Table 3). In particular, qualitative Doppler assessment might help in managing patients with a plateaued hCG. More importantly, evaluating UA Doppler indices seems to be a promising marker to predict methotrexate resistance. However, more studies are needed to fully understand the role of Doppler ultrasound in the prognostic risk evaluation of trophoblastic tumors.

Doppler ultrasound has several applications in evaluating patients with gestational trophoblastic disease. Its role in differentiating the type of molar pregnancy and predicting trophoblastic neoplasia might aid clinicians in the management and counseling of patients. However, more studies are needed to better evaluate these findings. Recent data also support the use of Doppler as an important ancillary tool in post-molar follow-up, particularly using UA PI values to predict methotrexate resistance.

## AUTHOR CONTRIBUTIONS

Lin LH, Bernardes LS and Hase EA performed the literature search and wrote the manuscript. Fushida K and Francisco RPV reviewed and corrected the manuscript.

## Figures and Tables

**Table 1:** Studies that evaluated the correlation between UA Doppler indices and the development of post-molar trophoblastic tumors before uterine evacuation. n: number of patients; S/D: systolic/diastolic velocity; PI: pulsatility index; RI: resistance index.

Study	Patients with spontaneous remission				Patients with persistent disease				Conclusion
		n	S/D	PI	RI	n	S/D	PI	RI	
Yalcin et al., 2001	16	5.1	1.82	0.8	5	2.27	0.86	0.55	Lower uterine artery indices (S/D, PI, RI) before uterine evacuation were associated with persistent disease (*p*<0.01).
Gungor et al., 1998	20	-	-	0.46	12	-	-	0.29	Lower uterine artery RI before uterine evacuation was associated with persistent disease (*p*<0.001).
Chan et al., 1996	11	-	-	0.76	21	-	-	0.69	No association between uterine artery RI before uterine evacuation and persistent disease.

**Table 2:** Studies that evaluated the UA RI in gestational trophoblastic disease, normal pregnancy and non-pregnant women. GTN: gestational trophoblastic neoplasia; HM: hydatidiform mole; n: number of patients; UA RI: uterine artery resistance index; CC: choriocarcinoma; IM: invasive mole; CHM: complete hydatidiform mole; PHM: partial hydatidiform mole.

Study	GTN		HM		Pregnant		Non-pregnant		Conclusion
	n	UA RI	n	UA RI	n	UA RI	n	UA RI	
Tepper et al., 1994	3	0.410 ± 0.04	-	-	20	0.494 ± 0.06	-	-	Lower UA RI in GTN compared with first-trimester pregnant woman
Chan et al., 1996	32	0.68 ± 0.16	-	-	18	0.8 ± 0.09	23	0.9 ± 0.08	Lower UA RI in GTN compared with non-pregnant and first-trimester pregnant woman
Hsieh et al., 1994	23	0.56 ± 0.19	15	0.75 ± 0.06	-	-	55	0.80 ± 0.05	Higher peak systolic velocity and lower UA RI in GTN compared with non-pregnant and post-evacuation uneventful moles
Zhou et al., 2005	32 (CC) 184 (IM)	0.25± 0.05 (CC) 0.28 ± 0.06 IM)	106 (CHM) 33 (PHM)	0.55 ± 0.06 (CHM) 0.56 ± 0.04 (PHM)	13	0.66 ± 0.05	-	-	Lower UA RI in GTN compared with hydatidiform moles and first-trimester pregnant woman

**Table 3:** Studies that evaluated the role of Doppler ultrasound to predict chemotherapy resistance. GTN: gestational trophoblastic neoplasia; n: number of patients; UA S/D: uterine artery systolic/diastolic velocity; UA PI: uterine artery pulsatility index; UA RI: uterine artery resistance index.

Study	Chemo-resistant GTN	Chemo-sensitive GTN	Conclusion
Park et al., 1994	5	11	UA S/D before treatment was not significantly different in patients with chemoresistant and sensitive disease. After completion of chemotherapy, UA S/D increased in patients with remitted disease (2.72±1.31 to 6.23±2.38), whereas in patients with resistant GTN, UA S/D did not change (2.69±1.8 to 3.08±1.54)
Oguz et al., 2004	7	30	A cuff-off value of 4 cm or higher of myometrial invasion and vascularization RI≤0.28 were proposed to predict single-agent chemotherapy resistance
Long et al., 1992	8	42	This prospective study established a cut-off value of UA PI≤1.1 associated with an elevated likelihood of GTN patients developing chemoresistance
Agarwal et al., 2002	47	117	This retrospective study established that a UA PI≤1.0 detected resistance with a sensitivity of 68% and specificity of 62%, with an adjusted odds ratio of 2.27 for methotrexate resistance in low-risk GTN
Sita-Lumsden et al., 2013	43	30	In patients with a GTN FIGO score of 5-6, the risk of chemoresistance was 67% with a UA PI≤1, compared with 42% when the UA PI was >1
Agarwal et al., 2012	113	126	A lower UA PI in patients with low-risk GTN with chemoresistance and UA PI≤1 was a risk factor independent of the FIGO score for methotrexate resistance. Patients with a FIGO score of 6 and UA PI≤1 had 100% resistance to methotrexate
Hsieh et al., 1994	13 (GTN needing ≥5 courses of chemotherapy)	10 (GTN needing <5 courses of chemotherapy)	Higher mean UA RI in patients requiring fewer than 5 cycles of chemotherapy (0.71±0.09) compared to patients requiring longer chemotherapy (0.47±0.14). There was an evident decrease in the UA peak systolic velocity after the first three chemotherapy cycles in the group requiring less courses of treatment (54.2 to 23.6 cm/s compared to 60.1 to 60 cm/s)
